# Gliding performance is affected by cranial movement of abdominal organs

**DOI:** 10.1038/s41598-020-78609-3

**Published:** 2020-12-08

**Authors:** Naoki Yoshida, Hideki Ota, Satoshi Higuchi, Yusuke Sekiguchi, Takaaki Kakihana, Haruka Sato, Tomoyoshi Kimura, Shin-Ichi Izumi, Masahiro Kohzuki

**Affiliations:** 1 Department of Internal Medicine and Rehabilitation Science, Tohoku University Graduate School of Medicine, 1-1 Seiryo-machi, Aoba-ku, Sendai, 980-8574 Japan; 2 Department of Diagnostic Radiology, Tohoku University Hospital, Sendai, Japan; 3 Department of Radiology, National Cerebral and Cardiovascular Center, Suita, Japan; 4 Department of Physical Medicine and Rehabilitation, Tohoku University Graduate School of Medicine, Sendai, Japan; 5 Department of Clinical Physiology, Tohoku University Graduate School of Medicine, Sendai, Japan; 6 Department of Physical Medicine and Rehabilitation, Tohoku University Graduate School of Biomedical Engineering, Sendai, Japan

**Keywords:** Magnetic resonance imaging, Anatomy

## Abstract

Swimming is an extremely popular sport around the world. The streamlined body position is a crucial and foundational position for swimmers. Since the density of lungs is low, the center of buoyancy is always on the cranial side and the center of gravity is always on the caudal side. It has been reported that the greater the distance between the centers of buoyancy and gravity, the swimmer’s legs will sink more. This is disadvantageous to swimming performance. However, the way to reduce the distance between the centers of buoyancy and gravity is yet to be elucidated. Here we show that swimmers with high gliding performance exhibit different abdominal cavity shapes in the streamlined body position, which causes cranial movement of the abdominal organs. This movement can reduce the distance between the centers of buoyancy and gravity, prevent the legs from sinking, and have a positive effect on gliding performance.

## Introduction

The streamlined body position is a crucial and foundational position for swimmers; furthermore, gliding ability plays an important role in race performance^1^. In the streamlined body position, the swimmer places one hand over the other, with their fingers overlapping, raises their arms above their head, straightens their legs, and plantar flexes their feet^2,3^. Swimming is one of the most challenging locomotion techniques for humans, and achieving a streamlined body position is important for improving swimming performance^4,5^. The glide phase occurs when the swimmer attempts to travel through the water while maintaining the streamlined body position and taking no other action^6^. One indicator of gliding performance is the distance traveled during glide intervals^6,7^. This is significantly affected by drag because swimming is performed in water, which has a greater density than air^1,4,8^. The drag force is referred to as “passive” if the participant is being towed or is gliding, with no limb movement, whereas it is considered “active drag” when the swimmer is propelling themself^5^. Hence, to improve gliding performance, passive drag must be reduced.

**Figure 1:**
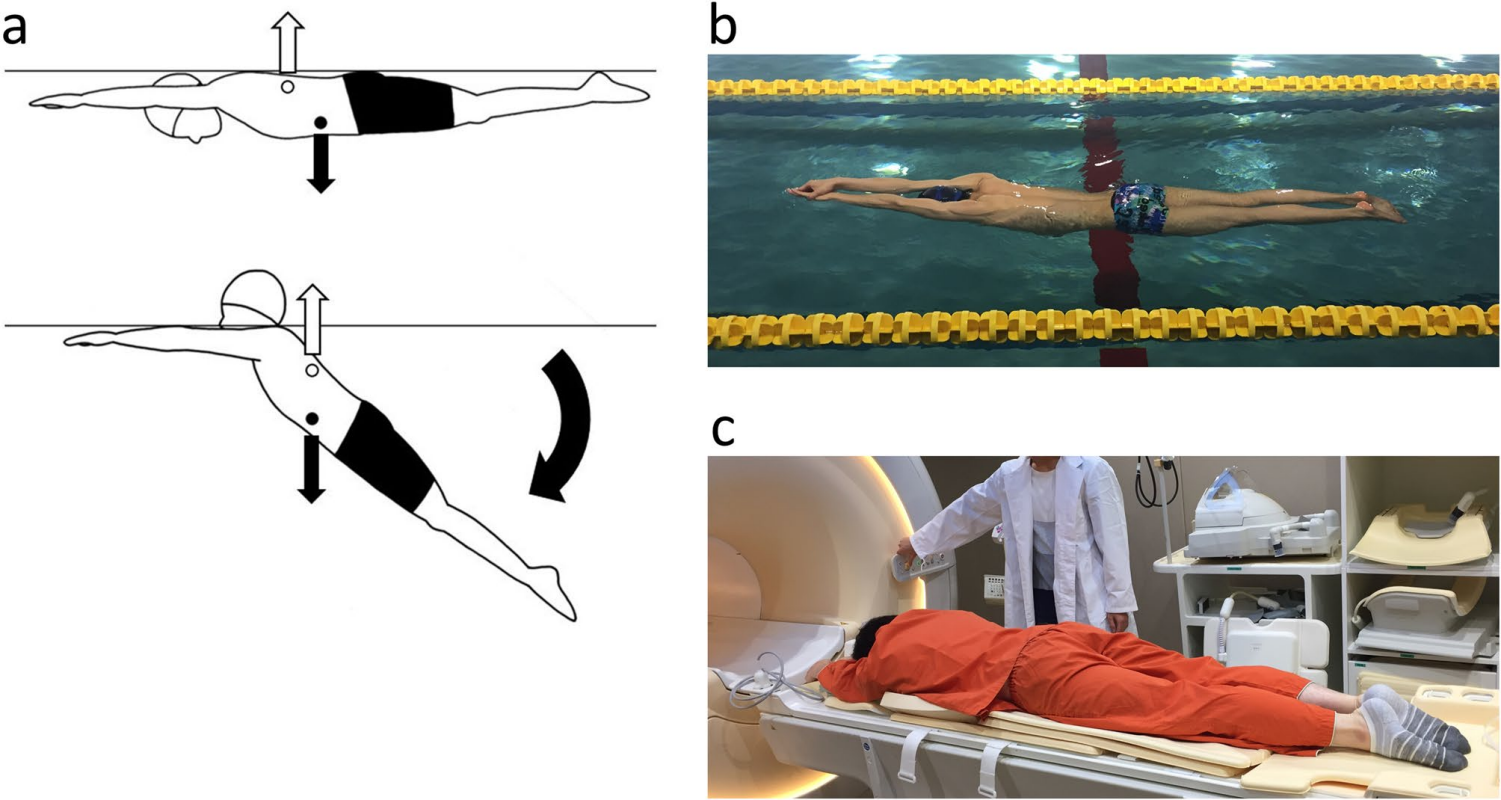
Streamlined body position. (**a**) Schematic of the streamlined body position in water. The white circle indicates the center of buoyancy, and the black circle indicates the center of gravity. The white arrow indicates buoyancy force, and black arrows indicate gravity force. Since lungs have lower density and are located in the chest, the center of buoyancy is always on the cranial side and the center of gravity is always on the caudal side. An object rotates in water until the center of buoyancy and the center of gravity are aligned vertically. As shown, sinking legs lead to increased frontal surface area. This is disadvantageous to swimming performance. (**b**) Glide is the phase in which the swimmer attempts to maintain speed in the streamlined body position without using actions to propel the body. (**c**) Participants were positioned in the prone position on the patient table.

The streamlined body position is acted upon by two forces in the water: buoyancy and gravity^9^. Because the chest contains the air-filled lungs, which have a lower density than the surrounding water, the center of buoyancy is located here, on the cranial side of the torso, whereas the center of gravity is always on the caudal side^10^. When submerged, an object will rotate until the centers of buoyancy and gravity are vertically aligned. In the human body, this rotation in the streamlined body position causes the legs to sink^11,12^ (Fig. 1a). This has been studied as underwater torque, which is one of the main factors increasing drag in swimming^12–14^. A previous study found that the greater the distance between the centers of buoyancy and gravity, the greater the underwater torque^15^. Thus, for reducing the effects of drag, investigating ways to reduce the distance between the centers of buoyancy and gravity in the streamlined body position is important. Some swimmers are able to keep their legs afloat in the streamlined body position without any significant action (e.g., kicking) (Supplementary Movie 1). As shown in the video, these swimmers use their abdominal muscles to draw in the belly, which in turn causes the legs to float. This phenomenon can be explained by abdominal contraction reducing the distance between the center of buoyancy and the center of gravity. Abdominal contraction may improve gliding performance because if the legs do not sink, the frontal surface area is reduced. As the frontal surface area decreases, the swimmer’s drag decreases^6,16^. However, the mechanism underlying the reduced distance between the centers of buoyancy and gravity when the belly is drawn in has not yet been elucidated.

The abdominal cavity is a large body cavity in humans and contains many organs. The average weights of abdominal organs in healthy males (18.5 kg/m^2^ ≤ body mass index (BMI) < 25 kg/m^2^) are as follows: liver, 1414 g (range 838–2013 g); right kidney, 121 g (range 84–200 g); left kidney, 129 g (range 93–201 g); and spleen, 127 g (range 53–299 g)^17^. The abdominal cavity also contains luminal organs, which have weights that are greatly affected by meals. It has been reported that these heavy abdominal organs can change their location in the abdomen depending on posture^18–21^. Hence, our hypothesis is that a swimmer with high gliding performance is able to move their abdominal organs to the cranial side of the abdominal cavity, thereby reducing the distance between the centers of buoyancy and gravity.

### MR examination for swimmers

**Figure 2:**
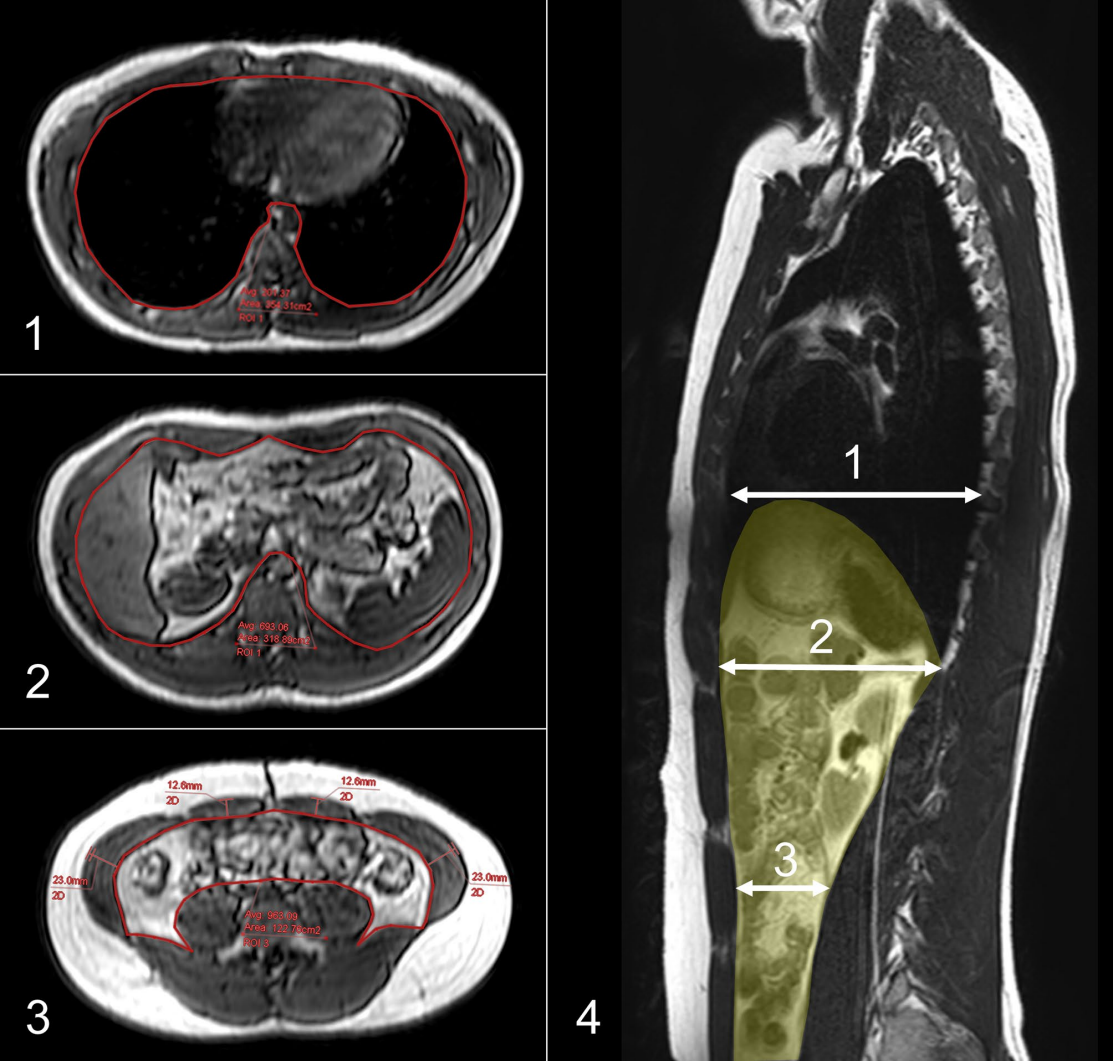
Measurements of the cross-sectional area of the abdominal cavity at three levels. **1** Axial MR image at the upper liver level showing measurements of cross-sectional area. **2** Axial MR image at the lower lung level showing measurements of cross-sectional area. **3** Axial MR image at the umbilical level showing measurements of cross-sectional area and thickness of the abdominal muscles. **4** Sagittal MR image at the left kidney level showing abdominal cavity, which is marked in yellow. (1) indicates the upper liver level, (2) indicates the lower lung level, and (3) indicates the umbilical level.

Seventeen male college swimmers participated in this study. The participants’ gliding distances in the streamlined body position were measured in a pool and used to divide participants into two groups (Fig. 1b). In the high performance group, the average gliding distance was > 10 m, whereas in the low performance group, the average gliding distance was < 10 m. No significant difference in characteristics with regard to age, height, weight, body mass index (BMI), body surface area (BSA), upper limb length, lower limb length, and shoulder width was noted between participants in the high performance group and those in the low performance group (Table 1). Participants were asked to lie in the prone position and MRI measurements were taken in resting and prescribed streamlined body positions (Fig. 1c). In order to examine the abdominal cavity shape, cross-sectional area (CSA) was measured at three levels along the torso: upper liver level, lower lung level, and umbilical level (Fig. 2).

**Table 1:** Participant characteristics.

	High performance group n = 8	Low performance group n = 9	P value
Gliding distance (m)	10.7 (10.4–13.1)	8.3 (8.0–9.0)	–
Age (years)	22.0 (21.8–23.0)	22.5 (20.8–23.3)	0.92
YSP (years)	11.0 (7.5–17.5)	4.0 (1.5–5.0)	0.012*
Height (m)	1.73 (1.71–1.77)	1.68 (1.62–1.77)	0.23
Weight (kg)	65.5 (61.5–70.3)	60.0 (52.0–67.0)	0.15
BMI (kg/m^2^)	21.1 (20.7–23.3)	20.3 (20.2–21.4)	0.28
BSA	1.78 (1.74–1.83)	1.68 (1.53–1.83)	0.17
Upper limb length (cm)	55.0 (54.5–56.5)	53.0 (50.0–57.0)	0.19
Lower limb length (cm)	89.5 (87.8–91.8)	84.0 (82.0–92.0)	0.25
Shoulder width (cm)	43.5 (42.0–45.0)	42.0 (42.0–43.0)	0.22

All data are presented as medians with interquartile ranges.

*YSP* the years of swimming practice, *BMI* body mass index, *BSA* body surface area.

*p < 0.05.

## Results

### CSA within-group comparisons

**Figure 3:**
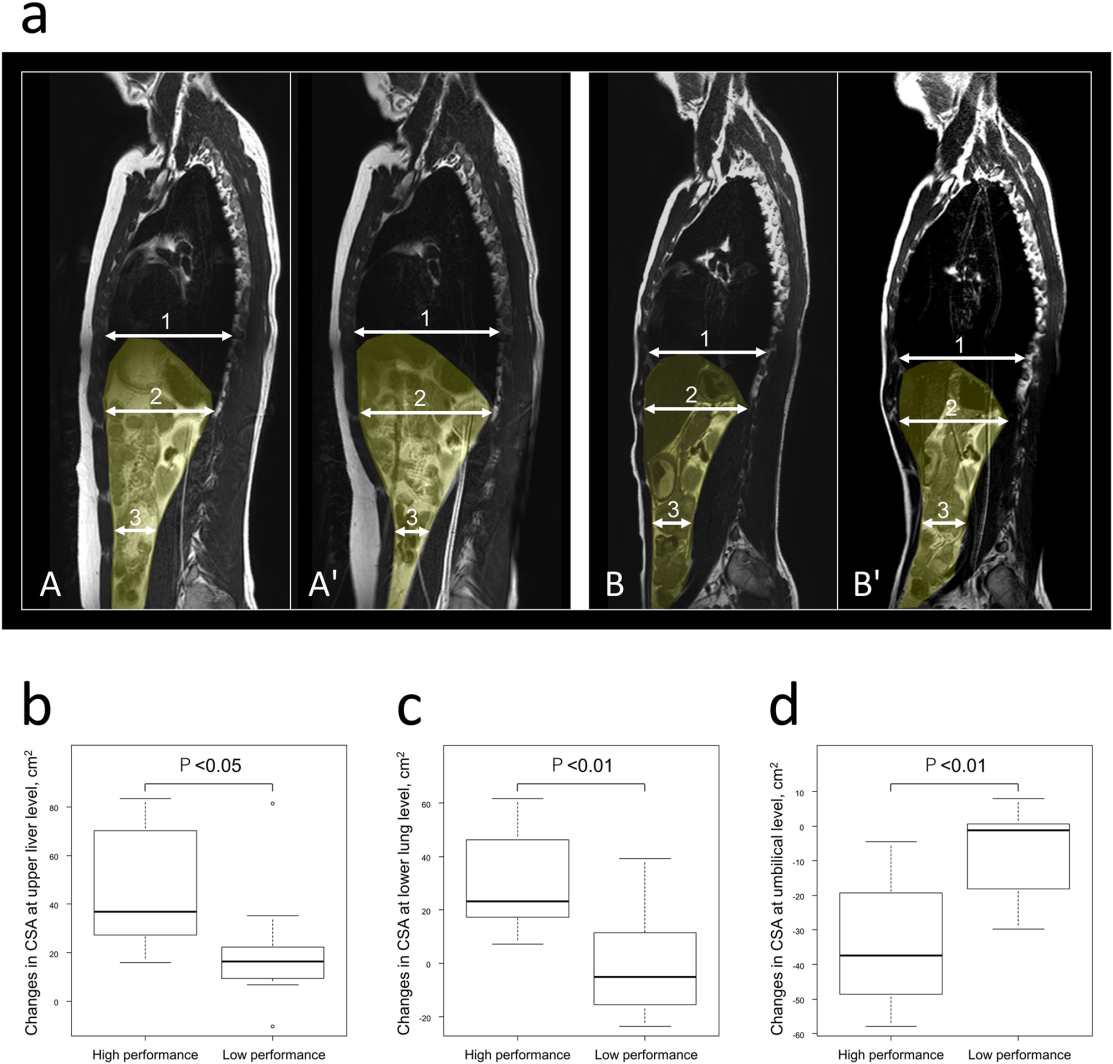
Representative case presentation and changes in CSA between study groups. (**a**) The sagittal MR images at the left kidney level show abdominal cavity, which is marked in yellow. (1) indicates the upper liver level, (2) indicates the lower lung level, and (3) indicates the umbilical level. (**a-A,A’**) Case 1 is a representative case of the high performance group: the years of swimming practice (YSP) 8 years, gliding distance 13 m. From the resting position (**A**) to the streamlined body position (**A’**). **a-B,B’**, Case 2 is the representative case for the low performance group: YSP 1 year, gliding distance 8 m. From the resting position (**B**) to the streamlined body position (**B’**). (**b**–**d**) Box plot of the changes in CSA between the high performance and low performance groups at three levels: upper liver level (**b**), lower lung level (**c**), umbilical level (**d**). Box plots show the median (center line), interquartile range (hinges), 10th and 90th percentiles (whiskers). CSA in the high performance group (as shown in Case 1: **a-A, A’**) increased at the upper liver and lower lung levels, and decreased at the umbilical level in the streamlined body position. These changes in CSA are significantly higher than those of the low performance group at all three levels (**b**–**d**).

In the high performance group, CSA increased significantly from the resting to the streamlined body positions at the upper liver and lower lung levels, and decreased significantly at umbilical level (Wilcoxon signed rank test, P < 0.01, P < 0.01, P < 0.01, respectively). In the low performance group, the CSA increased significantly only at the upper liver level, and there were no significant changes at the lower lung and umbilical levels (Wilcoxon signed rank test, P = 0.027, P = 0.65, P = 0.25, respectively) (Fig. 3a) (Table 2). Furthermore, we measured the maximum thickness of the rectus abdominis muscle (RA) and antero-lateral abdominal wall, which is composed of the transversus abdominis muscle (TrA), the external oblique muscle (EO), and internal oblique muscle (IO), at the umbilical level (TrA + EO + IO). In both high and low performance groups, the thickness of the TrA + EO + IO increased significantly from the resting to the streamlined body positions at the umbilical level (Wilcoxon signed rank test, P = 0.016, P = 0.012, respectively). However, in both the high and low performance groups, the thickness of the RA was not significantly altered (Wilcoxon signed rank test, P = 0.35, P = 0.25, respectively) (Table 2).

**Table 2:** Comparisons of CSA and thickness of abdominal wall muscle within groups.

	Resting position	Streamlined body position	P value
**CSA at upper liver level (cm**^**2**^**)**			
High performance group	327.3 (294.6–359.7)	392.8 (320.3–410.8)	< 0.01**
Low performance group	285.1 (277.7–308.8)	313.2 (305.4–335.6)	0.027*
**CSA at lower lung level (cm**^**2**^**)**			
High performance group	221.6 (211.9–262.0)	257.2 (241.9–306.0)	< 0.01**
Low performance group	221.7 (209.3–230.6)	239.0 (195.9–248.7)	0.65
**CSA at umbilical level (cm**^**2**^**)**			
High performance group	128.4 (107.7–135.2)	84.3 (70.2–94.9)	< 0.01**
Low performance group	110.8 (104.9–127.7)	106.6 (92.4–108.4)	0.25
**Thickness of rectus abdomen (mm)**			
High performance group	10.2 (9.1–13.0)	10.5 (9.3–14.1)	0.35
Low performance group	9.2 (7.9–9.4)	9.6 (8.5–11.5)	0.25
**Thickness of TrA + EO + IO (mm)**			
High performance group	20.6 (19.0–21.6)	27.1 (22.2–29.6)	0.016*
Low performance group	18.7 (17.2–20.1)	19.6 (18.6–23.8)	0.012*

All data are presented as medians with interquartile ranges.

*CSA* cross-sectional area, *TrA* transversus abdominis muscle, *EO* external oblique muscle, *IO* internal oblique muscle.

*p < 0.05, **p < 0.01.

### Changes in CSA between study groups

In the high performance group, changes in CSA from resting to streamlined body positions were: at upper liver level 36.8 cm^2^ (median, interquartile range 28.7 to 67.3), at lower lung level 23.2 cm^2^ (17.3–44.2), at umbilical level − 37.4 cm^2^ (− 47.4 to − 24.9). In the low performance group, the following changes occurred in the CSA when transitioning from the resting to streamlined body positions: 16.4 cm^2^ (9.3–22.2) at the upper liver level, − 5.1 cm^2^ (− 15.4 to 11.6) at the lower lung level, and − 1.2 cm^2^ (− 18.1 to 0.75) at the umbilical level. The changes in the CSA were significantly greater in the high performance group than in the low performance group at all three levels: upper liver level, lower lung level, umbilical level (Mann–Whitney U test, P = 0.036, P < 0.01, P < 0.01, respectively) (Fig. 3b–d) (Table 3). These change in abdominal cavity shape can cause cranial side movement of abdominal organs.

**Table 3:** Changes in CSA from resting to streamlined body position at three levels between two group.

	High performance group	Low performance group	P value
Changes of CSA at upper liver level (cm^2^)	36.8 (28.7 to 67.3)	16.4 (9.3 to 22.2)	0.036*
Changes of CSA at lower lung level (cm^2^)	23.2 (17.3 to 44.2)	− 5.1 (− 15.4 to 11.6)	< 0.01**
Changes of CSA at umbilical level (cm^2^)	− 37.4 (− 47.4 to − 24.9)	− 1.2 (− 18.1 to 0.75)	< 0.01**

All data are presented as medians with interquartile ranges.

*CSA* cross-sectional area.

*p < 0.05, **p < 0.01.

### Representative case presentation

In order to compare the characteristics of the high and low performance groups, we present two cases. Case 1 is the representative case for the high performance group: height 1.80 m, weight 79 kg, the years of swimming practice (YSP) 8 years, and gliding distance 13 m. From the resting position to the streamlined body position, CSA at the upper liver level changed from 354.3 to 437.9 cm^2^, CSA at the lower lung level changed from 318.9 to 326.2 cm^2^, CSA at the umbilical level changed from 122.8 to 71.7 cm^2^ (Fig. 3a-A,A'). Case 2 is the representative case for the low performance group; height 1.67 m, weight 60 kg, YSP 1 year, and gliding distance 8 m. From the resting position to the streamlined body position, CSA at the upper liver level changed from 303.9 to 313.2 cm^2^, CSA at the lower lung level changed from 230.6 to 245.1 cm^2^, and CSA at the umbilical level changed from 113.8 to 112.5 cm^2^ (Fig. 3a-B,B'). Swimmers with high performance group (as shown in Case 1) exhibit different abdominal cavity shapes in the streamlined body position compared to low performance group (as show in Case 2), which can cause cranial-side movement of abdominal organs.

## Discussion

### Cranial-side movement of abdominal organs

To our knowledge, this is the first study to analyze the change in abdominal cavity shape in the streamlined body position by MRI. Results indicated that the high performance group experienced significant changes in CSA in the streamlined body position compared to those of the low performance group. Specifically, CSA of the high performance group increased at the upper liver and lower lung levels, and decreased at the umbilical level in the streamlined body position. This change in abdominal cavity shape may cause cranial-side movement of abdominal organs (Fig. 3a). When these abdominal organs move to the cranial side in the streamlined body position, the center of gravity accordingly shifts to the cranial side.

### Center of gravity

The center of gravity is an imaginary point through which the gravitational force acts on an object^22^. In order to objectively measure the movement of the center of gravity from the resting to the streamlined body positions, we further recruited 10 from the 17 participants who underwent MR imaging. Five participants were the high performance group, and other five participants were the low performance group. No significant differences in characteristics with regard to height, the length between the tip of the longest finger and the soles of the feet, and BMI were observed between participants in the high performance group and those in the low performance group (Table 4). The center of gravity was measured using the reaction board method^10,23^, and ground reaction force data were acquired using 90 × 60 cm force plates (Anima Corporation, Chofu, Tokyo, Japan)^24^. The participants were placed on the balance board in the supine position, and measurements were performed in both the streamlined body position and resting position. For the streamlined body position, participants were instructed to hold a streamlined position as if underwater. For the resting position, participants were instructed to raise their arms above their head, like in the streamlined body position, and to relax their body.

**Table 4:** Participant characteristics in the measurement of center of gravity.

	High performance group n = 5	Low performance group n = 5	P value
Gliding distance (m)	10.6 (10.5–10.8)	8.3 (8.0–9.0)	–
Height (m)	1.70 (1.69–1.74)	1.65 (1.61–1.68)	0.059
Length between the tip of the longest finger and the soles of the feet (m)	2.11 (2.09–2.18)	2.04 (2.01–2.10)	0.12
Weight (kg)	68.7 (67.4–73.4)	59.8 (48.8–62.3)	0.032*
BMI (kg/m^2^)	22.3 (22.2–24.3)	21.2 (19.1–22.9)	0.22
CGx% (%)	0.334 (0.184–0.359)	0.079 (0.048–0.170)	0.032*

Data are presented as medians with interquartile ranges.

CG is the distance from the feet to the participant’s center of gravity. CGx is the movement of CG from the resting position to the streamlined body position. CGx% is the ratio of CGx to the length between the tip of the longest finger and the soles of the feet.

*p < 0.05.

The measurement point was at the tip of the finger, and the pivot point was at the soles of the feet (Supplementary Fig. 1). The center of gravity was calculated as follows:

1 $$CG= \frac{L \times RF}{W}$$

where CG is the distance from the feet to the participant’s center of gravity, L is the length between the tip of the longest finger and the soles of the feet, RF is the reaction force at the tip of the finger (without the weight of the balance board itself), and W is the participant’s weight. The movement of CG from the resting position to the streamlined body position (CGx) was calculated as follows:

2 $$CGx = CG1 - CG2 = \frac{L \times (RF1 - RF2)}{W}$$

where CG1 is the center of gravity in the streamlined body position, CG2 is the center of gravity in the resting position, RF1 is the center of gravity in the streamlined body position, and RF2 is the center of gravity in the resting position. To perform comparisons between the participants, CGx was expressed as a percentage (CGx%) of each participant’s length between the tip of the longest finger and the soles of the feet as follows:

3 $$CGx\% = \frac{100 \times CGx}{L}$$

The median CGx% was 0.334 (interquartile range 0.184–0.359) in the high performance group and 0.079 (0.048–0.170) in the low performance group. CGx% was significantly greater in the high performance group compared with the low performance group (Mann–Whitney U test, P = 0.032) (Table 4). The CGx% was a positive value, which means that the center of gravity moved cranially when the participants were instructed to assume the streamlined body position. Furthermore, the movement of the center of gravity was significantly greater in the high performance group. These results are consistent with our MRI findings and underpin our hypothesis. In other words, the cranial movement of the center of gravity is caused by the cranial movement of the abdominal organs and activated abdominal muscles. This movement of center of gravity reduces the distance between the centers of buoyancy and gravity, causing decreased underwater torque.

### Underwater torque

Underwater torque is one of the main effects of increasing the frontal surface area in the streamlined body position^25^. As increased frontal surface area leads to increased drag, swimmers aim to reduce their underwater torque^26^. The center of buoyancy in the human body is always on the cranial side, and the center of gravity is always on the caudal side^10^. The distance between the centers of buoyancy and gravity varies from individual to individual and depends on factors such as age, gender, and body composition^9,16,26^. Usually, the distance is greater for males than females^12^ and for adults compared with children^14,27^. In body composition studies, the density of the fat component is assumed to be approximately 0.9007 g/cm^3^ and that of fat-free muscle is approximately 1.066 g/cm^3^ ^28^. Because water density is 1.00 g/cm^3^, fat will float and muscle will sink. When the fat component is concentrated in the lower body, the distance between the centers of buoyancy and gravity decreases^15^. The use of buoyancy tools such as pull buoys (which are widely used as swimming tools around the world) also affects underwater torque. Swimmers place the pull buoy between their thighs or their ankles while swimming and the pull buoy reduces underwater torque by providing improved flotation support for hips and legs^15^. However, swimmers are not allowed to use support tools in competitions. It is impossible to change the age of a swimmer, and impractical to alter gender, and body composition cannot be changed within a short period of time. Hence, it is imperative that swimmers find other techniques to reduce underwater torque.

### Swimmer’s drag and body surface area

Underwater torque increases the frontal surface area, which causes the swimmer’s drag to increase. In addition to the increasing frontal surface area due to underwater torque, the BSA is also an important factor affecting a swimmer’s drag^3,4^. Frictional drag, which is one type of swimmers’ drag, can be calculated from the density of the water, the gliding velocity, and the BSA by numerical simulations (computer fluid dynamics, CFD)^5,29^. The BSA was calculated using Du Bois formulas^30^ based on height and weight. In our study, there was no significant difference in the BSA between participants in the high performance group and those in the low performance group.

### Abdominal drawing-in maneuver

In our study, the thickness of the antero-lateral abdominal wall was increased and CSA was decreased at the umbilical level in the streamlined body position. This action is similar to the abdominal drawing-in maneuver (ADIM), a body trunk exercise. The ADIM is a method which increases abdominal pressure by pulling the abdominal walls to the inside that the TrA and oblique abdominal muscles are contracted^31^. Because lumbar trunk stability is effectively accomplished through the increase in abdominal pressure, the ADIM has been reported to be important in the rehabilitation of lower back pain^32,33^. The action of the abdominal wall muscles during ADIM has been studied using ultrasound imaging, MRI, and electromyography^34,35^. The muscle bellies of the antero-lateral abdominal wall are observed to thicken and shorten in length during the ADIM, whereas the RA muscle does not thicken during the ADIM^34,36^. These muscle thickening responses were also observed in our study. From the resting position to the streamlined body position, the thickness of the antero-lateral abdominal wall muscle increased significantly, whereas the thickness of the RA muscle did not change significantly. A previous study also reported that the CSA of the trunk (including the abdominal cavity and trunk muscles but excluding subcutaneous tissue) at the L3–L4 disc decreased with the ADIM^32,34^. This CSA differed from our study in the point of measurement range and measurement height; however, the observed decrease in the CSA at the umbilical level was also observed in our study. Therefore, despite being instructed to only hold the streamlined position, it appears that these participants also unconsciously made similar movements to the ADIM. Since abdominal contraction, similar to ADIM, occurred unconsciously, it was impossible to assess the gliding distance both with and without abdominal contraction.

### Limitations

There are several limitations to this study. First, the streamlined body position in MRI may not be exactly the same as the streamlined body position in water. However, it is impossible to take MR images in water because of the interference caused by water. To ameliorate this, we used a soft cushion on the ventral side so that participants could hold the streamlined body position easily, and checked the body position through the monitor during MRI scans. Second, while swimming, swimmers do not hold their breath but breath out normally under water. This differs from the glide phase performed in our study. We measured the maximum gliding distance and had the participants hold their breath while gliding. Thus, our study results based on this glide may not be completely applicable to normal swimming. Third, although gliding distance is mainly influenced by drag, other factors such as physique, push-off characteristics, or swimsuits also may have an effect^37–39^. In this sense, some precaution should be taken when interpreting gliding performance because it can have some biases^40^. Our participants were college swimmers, so they were not beginner and had a certain amount of swimming ability. There was no physical difference between the two groups. They all wore standard swimsuits, rather than high-performance swimsuits. Therefore, we assumed that these factors are comparable between the two groups in this study.

## Conclusion

Swimmers with high gliding performance exhibit different abdominal cavity shapes in the streamlined body position compared to low performance group, which causes cranial movement of abdominal organs. This movement may reduce underwater torque, prevent the legs from sinking during swimming, and have a positive effect on gliding performance.

## Methods

This prospective study was conducted at Tohoku University Hospital in Japan. The study was approved by the ethics committee of Tohoku University (approval number: 15263), and all procedures were performed in accordance with the approved guidelines. Written informed consent was obtained from all participants. Written informed consent was obtained from the swimmer in Supplementary Movie 1 for publication in an online open-access publication.

### Participants

In this prospective cohort study, we consecutively recruited healthy, male college swimmers between 20 and 30 years old, with BMI of 18–25. Swimmers were excluded if they experienced any pain in the streamlined body position, had neurological or respiratory disorders, had contraindications to MRI, such as claustrophobia, or were deemed by doctors as inappropriate for study participation. Seventeen male college swimmers participated in this study.

### Measurements on land

The height, weight, upper extremity length, lower extremity length, and shoulder width of each participant was measured by an experienced physiotherapist. Upper extremity length was measured from the acromion process to the tip of the radial styloid process. Lower extremity length was measured from the anterior superior iliac spine to the medial malleolus. Shoulder width was measured between the acromion processes. BSA was calculated using Du Bois formulas^30^ based on height and weight.

### Gliding distance

The gliding distance in the streamlined body position was measured for each participant in an indoor pool (Fig. 1b). Gliding distance was defined as the maximum distance they could cover in the streamlined body position without any arm strokes or kicks. The pool was 25 m long, and the depth of the pool was 1.3 m. Participants used standard swimsuits. They began by standing on the pool floor, then submerged and maximally pushed off the wall in the streamlined body position. The distance between the pool wall and the tip of the participant’s hand was accurately measured when gliding ceased. This measurement was performed five times for each participant and the average measurement was used for sorting participants into two groups for comparison: the high performance group, in which average gliding distance was > 10 m, and the low performance group, in which average gliding distance was < 10 m.

### MR examination

MR examinations were performed using a 3.0-T whole-body MR scanner (Ingenia 3.0 T, Philips Medical Systems, Best, the Netherlands) with a 32-channel torso coil. Participants were placed in the prone position with their hands up and overlapping, head between the extended arms, feet together and plantar flexed (Fig. 1c). A soft cushion was used on the ventral side so that the prone position could be held without difficulty. MR scans were performed in both the resting and streamlined body positions.

*Streamlined body position* Participants were instructed to hold the streamlined body position as if underwater, inhale, and hold their breath.

*Resting position* Participants were instructed to relax their body, inhale the same amount of air as they had in the streamlined body position and hold their breath.

The volume of air-intake was determined by the participants, provided that air intake in the resting position was equal to that in the streamlined body position using auto voice. Two-dimensional (2D) turbo-spin echo T2-weighted images were acquired in sagittal orientations with repetition time (TR) = 2441 ms, echo time (TE) = 135 ms, flip angle (FA) = 90°, slice thickness = 4 mm, acquisition matrix = 380 × 225, field of view = 38 cm × 38 cm. Three-dimensional (3D) T1-weighted fast-field echo (enhanced T1 high-resolution isotropic volume excitation [eTHRIVE, Philips Medical Systems]) images were acquired in coronal orientations with TR = 3.7 ms, TE = 1.8 ms, FA = 10°, slice thickness = 4 mm, field of view = 50 cm × 50 cm. 2D T2-weighted and 3D T1-weighted images were acquired to cover the body trunk in two stations. Each MRI scan was performed within 20 s, during which the participants held their breath. The multi-station images were combined into a single full-field view on the MR console (MR MobiView, Philips Medical Systems).

### MR image analysis

The MR images were analyzed using a commercially available workstation (Ziostation2; Ziosoft, Tokyo, Japan). The acquired T1-weighted images were reconstructed into axial images. In order to evaluate the cranial movement of abdominal organs, CSA of the abdominal cavity was measured at three levels: upper liver level, lower lung level, and umbilical level. In this study, retroperitoneal space was included in CSA, but the aorta and inferior vena cava were excluded because they were relatively fixed. Furthermore, we measured the maximum thickness of the RA and antero-lateral abdominal wall, composed of the TrA and the EO and IO, at umbilical level (TrA + EO + IO). Thicknesses of muscles were measured on both right and left sides, and an average value for each was calculated (Fig. 2).

### Statistical analysis

All statistical analyses were performed with EZR (Saitama Medical Center, Jichi Medical University, Saitama, Japan)^41^, which is a graphical user interface for R (The R Foundation for Statistical Computing, Vienna, Austria). More precisely, it is a modified version of the R commander designed to add statistical functions frequently used in biostatistics. All continuous variables were presented as medians with interquartile ranges. The Mann–Whitney U test was used for between-group comparisons, and the Wilcoxon signed rank test was used for within-group comparisons. All P-values of < 0.05 were considered statistically significant.

## Supplementary Information

Supplementary Video 1.

Supplementary Information 1.

Supplementary Information 2.

Supplementary Information 3.

## Data Availability

The data that support the findings of this study are available from the corresponding author upon reasonable request.
