# Supplementary material for: Gliding performance is affected by cranial movement of abdominal organs
Source: Sci Rep. 2020 Dec 8;10:21430. doi: 10.1038/s41598-020-78609-3 (PMC7722763; doi:10.1038/s41598-020-78609-3)
Supplement: Supplementary file 2 — Supplementary Information 1. [file 41598_2020_78609_MOESM2_ESM.docx]

**TITLE**

**Gliding performance is affected by cranial movement of abdominal organs**

**AUTHORS**

Naoki Yoshida^1*^, Hideki Ota^2^, Satoshi Higuchi^2, 3^, Yusuke Sekiguchi^4^, Takaaki Kakihana^1^, Haruka Sato^5^, Tomoyoshi Kimura^2^, Shin-Ichi Izumi^4, 6^, Masahiro Kohzuki^1^.

**AFFILIATIONS**

^1^Department of Internal Medicine and Rehabilitation Science, Tohoku University Graduate School of Medicine, Sendai, Japan

^2^Department of Radiological Technology, Tohoku University Hospital, Sendai, Japan

^3^Department of Radiology, National Cerebral and Cardiovascular Center, Suita, Japan

^4^Department of Physical Medicine and Rehabilitation, Tohoku University Graduate School of Medicine, Sendai, Japan

^5^Department of Clinical Physiology, Tohoku University Graduate School of Medicine, Sendai, Japan

^6^Department of Physical Medicine and Rehabilitation, Tohoku University Graduate School of Biomedical Engineering, Sendai, Japan

*Correspondence to: Naoki Yoshida, 1-1 Seiryo-machi, Aoba-ku, Sendai, Japan, 980-8574, TEL: +81-22-717-7353, FAX: +81-22-717-7355, [nyoshida.thk@gmail.com](mailto:nyoshida.thk@gmail.com)

**LEGENDS**

**Supplementary Movie 1**

The video shows a swimmer floating his legs in the streamlined body position by merely drawing in his abdominal muscles. His legs float without the use of any tools, propulsion, or any other remarkable action like underwater kicking. The drawing-in belly maneuver causes cranial movement of abdominal organs, and reduces the distance between the centers of buoyancy and gravity, reducing underwater torque.
