# Supplementary figures and images for: Gliding performance is affected by cranial movement of abdominal organs
Source: Sci Rep. 2020 Dec 8;10:21430. doi: 10.1038/s41598-020-78609-3 (PMC7722763; doi:10.1038/s41598-020-78609-3)

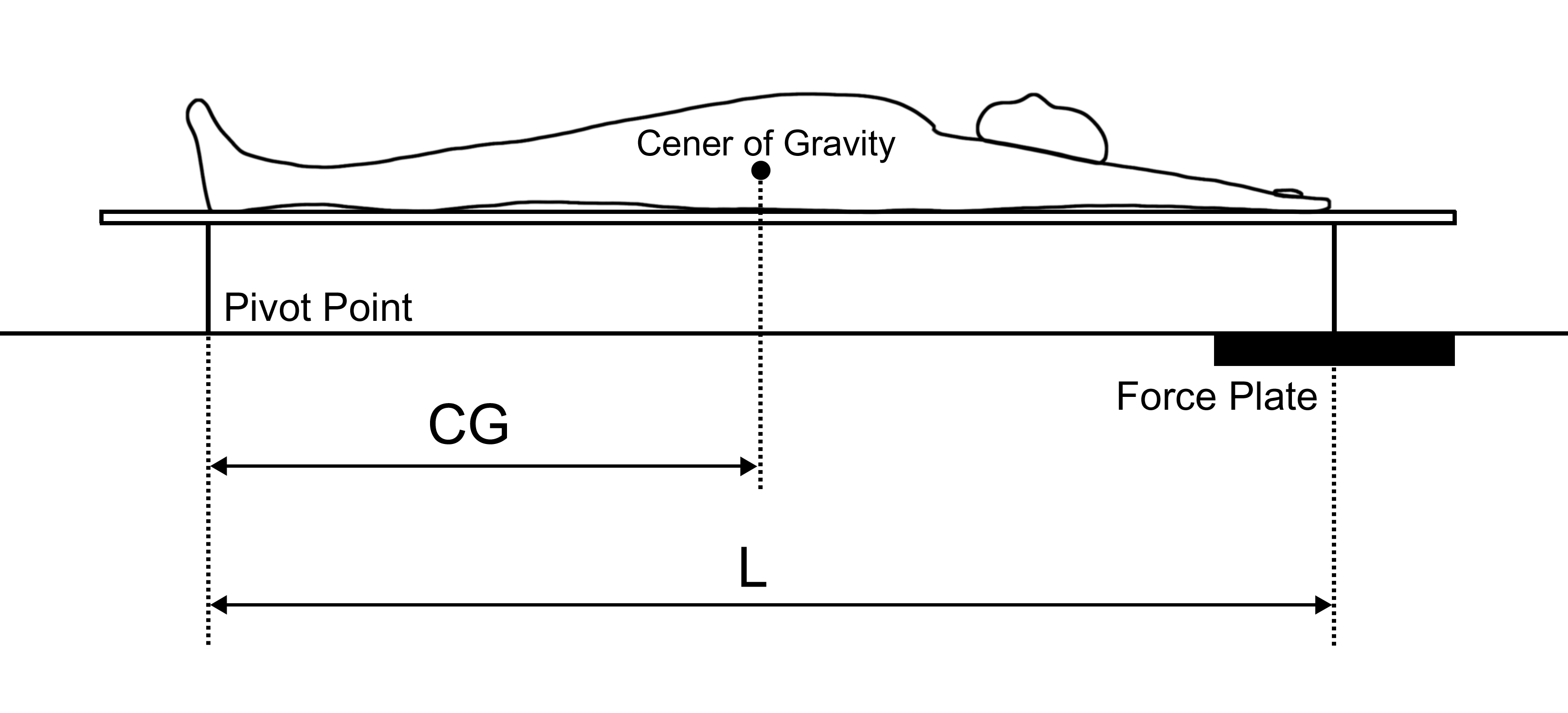

Supplement: Supplementary file 3 — Supplementary Information 2. [file 41598_2020_78609_MOESM3_ESM.tif]
