# Supplementary material for: Gliding performance is affected by cranial movement of abdominal organs
Source: Sci Rep. 2020 Dec 8;10:21430. doi: 10.1038/s41598-020-78609-3 (PMC7722763; doi:10.1038/s41598-020-78609-3)
Supplement: Supplementary file 4 — Supplementary Information 3. [file 41598_2020_78609_MOESM4_ESM.docx]

**TITLE**

**Gliding performance is affected by cranial movement of abdominal organs**

**AUTHORS**

Naoki Yoshida^1*^, Hideki Ota^2^, Satoshi Higuchi^2, 3^, Yusuke Sekiguchi^4^, Takaaki Kakihana^1^, Haruka Sato^5^, Tomoyoshi Kimura^2^, Shin-Ichi Izumi^4, 6^, Masahiro Kohzuki^1^.

**AFFILIATIONS**

^1^Department of Internal Medicine and Rehabilitation Science, Tohoku University Graduate School of Medicine, Sendai, Japan

^2^Department of Radiological Technology, Tohoku University Hospital, Sendai, Japan

^3^Department of Radiology, National Cerebral and Cardiovascular Center, Suita, Japan

^4^Department of Physical Medicine and Rehabilitation, Tohoku University Graduate School of Medicine, Sendai, Japan

^5^Department of Clinical Physiology, Tohoku University Graduate School of Medicine, Sendai, Japan

^6^Department of Physical Medicine and Rehabilitation, Tohoku University Graduate School of Biomedical Engineering, Sendai, Japan

*Correspondence to: Naoki Yoshida, 1-1 Seiryo-machi, Aoba-ku, Sendai, Japan, 980-8574, TEL: +81-22-717-7353, FAX: +81-22-717-7355, [nyoshida.thk@gmail.com](mailto:nyoshida.thk@gmail.com)

**LEGENDS**

**Supplementary Figure 1**

Schematic of the reaction board method used for measuring the center of gravity. The black circle indicates the center of gravity, and the black rectangle indicates the force plate. CG is the distance from the feet to the participant’s center of gravity, and L is the length between the tip of the longest finger and the soles of the feet. The measurement point was at the tip of the finger, while the pivot point was at the soles of the feet. We used the force plate to measure the reaction force at the tip of the finger (without the weight of the balance board itself).
